# Case Report: Evolution of a Severe Vascular Refractory Form of ECD Requiring Liver Transplantation Correlated With the Change in the Monocyte Subset Analysis

**DOI:** 10.3389/fimmu.2021.755846

**Published:** 2021-11-12

**Authors:** Jérôme Razanamahery, Anne Roggy, Jean-François Emile, Alexandre Malakhia, Zaher Lakkis, Francine Garnache-Ottou, Thibaud Soumagne, Fleur Cohen-Aubart, Julien Haroche, Bernard Bonnotte

**Affiliations:** ^1^ Department of Internal Medicine and Clinical Immunology, Francois Mitterrand Hospital, Dijon University Hospital, Dijon, France; ^2^ Établissement Français du Sang Bourgogne Franche-Comté, Laboratoire d’Hématologie et d’Immunologie Régional, Besançon, France; ^3^ Department of Pathology, Ambroise-Paré Hospital, Assistance-Publique Hopitaux de Paris, Paris, France; ^4^ Department of Radiology, Francois Mitterrand Hospital, Dijon University Hospital, Dijon, France; ^5^ Digestive Surgery Unit, University of Bourgogne Franche-Comté, Centre Hospitalier Regional Universitaire (CHRU) Besançon, Besancon, France; ^6^ Department of Intensive Care Unit, Besancon University Hospital, Besancon, France; ^7^ Internal Medicine Department 2, National Reference Center for Histiocytosis, Pitié-Salpêtrière Hospital, Assistance Publique Hôpitaux de Paris, Paris, France

**Keywords:** monocyte, histiocytosis, Erdheim–Chester disease, transplantation, vascular diagnosis

## Abstract

Erdheim–Chester disease is a rare histiocytosis characterized by iconic features associated with compatible histology. Most patients have somatic mutations in the MAP-kinase pathway gene, and the mutations occur in CD14^+^ monocytes. Differentiation of the myeloid lineage plays a central role in the pathogenesis of histiocytosis. Monocytes are myeloid-derived white blood cells, divided into three subsets, but only the CD14^++^CD16^−^ “classical monocyte” can differentiate into dendritic cells and tissue macrophages. Since most mutations occur in CD14^+^ cells and since ECD patients have a particular monocytic phenotype resembling CMML, we studied the correlation between disease activity and monocytic subset distribution during the course of a severe vascular form of ECD requiring liver transplantation. During early follow-up, increased CD14^++^CD16^−^ “classical monocyte” associated with decreased CD14^low^CD16^++^ “non-classical monocyte” correlated with disease activity. Further studies are needed to confirm the use of monocyte as a marker of disease activity in patients with ECD.

## Highlights

- This case report raises the question of whether an increase in CD14^++^CD16^−^ “classical monocytes” associated with a decrease in CD14^low^CD16^++^ “non-classical monocytes” correlates with ECD activity.
- Monocyte immunophenotyping could be a simple and reproductible tool to assess disease activity in ECD patients.
- Monocyte immunophenotyping can be repeated frequently unlike metabolic evaluation

## Introduction

Monocytes are myeloid-derived white blood cells divided into three subsets (classical, intermediate, non-classical) based on the level of expression of surface chemokines (1) (CD14 and CD16). The functions of the three subsets (classical, intermediate, and non-classical) are different, but only the classical monocyte can differentiate into dendritic cells and tissue macrophages (2, 3).

The involvment of myeloid lineage differentiation, particularly monocytes, has been highlighted in neoplasia and plays a central role in the genesis of histiocytoses (4). Histiocytoses are orphan diseases characterized by the proliferation of dendritic cells and various monocyte-macrophage (histiocytic) cell types infiltrating tissues and causing organ damage (5). Among the histiocytoses, Erdheim–Chester disease (ECD) is a rare clonal histiocytosis characterized by iconic features (long bone, retroperitoneal, and vascular involvement) associated with compatible histology (CD68+, CD1a−, S100− histiocyte infiltration with various degrees of fibrosis) (6). In most biopsies, histiocytes express a phosphorylated extracellular signal-regulated kinase (p-Erk) testifying mitogen-activated protein kinase (MAP-kinase) pathway gene activation (7, 8). This activation of p-ERK is the final nuclear traduction of somatic mutations on the MAP-kinase pathway genes present in most ECD patients (6). Few other patients harbor a mutation in *PI3K/AKT/mtor* pathway, and unmutated patients represent <15% of ECD patients (9). Mutations in the MAP-kinase pathway genes (particularly *BRAF*) occur mainly in CD14^++^ CD16^low^ monocytes (10, 11).

Treatment is based on conventional agents [pegylated interferon, mammalian target of rapamycin (mTOR) inhibitors], biological agents [interleukin (IL)-1 or tumor necrosis factor (TNF)-alpha inhibitors], or targeted therapies (*BRAF/MEK* inhibitors) depending on staging and molecular status (6). Staging evaluation is based on metabolic response using PRECIST criteria (12) by analogy to solid tumors. However, these assessments, repeated every 6 months or every year, are not accurate in deciphering the specific activity of ECD in relation to infectious or other inflammatory processes.

To date, no biological marker of the disease activity has been so far validated.

Papo et al. recently demonstrated that a particular monocyte phenotype, resembling chronic myelomonocytic leukemia (CMML), was found in ECD patients (13). Moreover, CD14^++^ CD16^low^ monocytes play a central role in ECD pathogenesis, as they carry somatic mutations in most cases (10, 11). Based on these points, our case report presents (14) a correlation between clinical activity and monocytes subset distribution during the follow-up of a patient presenting a severe vascular ECD requiring liver transplantation.

## Case Description

We report a case of a patient with a severe vascular form of ECD requiring liver transplantation for whom a correlation between monocytes subset distribution and disease activity was shown.

## Methods

Histology was performed on 4-μm thick tissue sections after staining with hematoxylin & eosin and immunohistochemistry, including at least CD1a, S100, and (CD68, CD163) primary antibodies (7). Tumor DNA was extracted from formalin-fixed and paraffin-embedded tissues. Detection of MAP-kinase pathway genes’ mutations was performed using targeted next-generation sequencing. Samples were analyzed using MiSeq (Illumina) after preparing the Custom Amlicon Low Input Kit libraries. The targeting genes were *AKT1*, *ALK*, *ARAF*, *ASXL1*, *BRAF*, *CALR*, *CBL*, *CDK4*, *CDKN1B*, *CDKN2A*, *CEBPA*, *CSF3R*, *CTNNB1*, *DNMT3A*, *EGFR*, *EZH2*, *FLT3*, *GATA2*, *GNA11*, *GNAQ*, *GNAS*, *HERC1*, *HRAS*, *IDH1*, *IDH2*, *JAK2*, *JAK3*, *KIT*, *KRAS*, *KTM2D*, *MAML3*, *MAMLD1*, *MAP2K1*, *MAP2K2*, *MAP2K3*, *MAP2K4*, *MAP2K6*, *MAP3K1*, *MAP3K8*, *MAP3K9*, *MAP3K10*, *MAP3K19*, *MAP4K4*, *MAPK1*, *MAPK11*, *MAPK9*, *MPL*, *NF1*, *NOTCH1*, *NOTCH2*, *NPM1*, *NRAS*, *PDGFRA*, *PIK3CA*, *PP6C*, *PTEN*, *PTPN11*, *RAC1*, *RAF1*, *RIT1*, *RUNX1*, *SETBP1*, *SRSF2*, *STAG2*, *STK19*, *SYNGAP1*, *TAOK1*, *TAOK2*, *TET2*, *TP53*, *U2AF1*, *WT1*, and *ZRSR2* as described by Melloul et al. (15)

The gene panel analyzed by next-generation sequencing (NGS) on bone marrow were *ASXL1*, *BCOR*, *BCORL1*, *CALR*, *CBL*, *CSF3R*, *DNMT3A*, *ETV6*, *EZH2*, *FLT3, GATA2, IDH1, IDH2*, *JAK2*, *KIT*, *KRAS*, *MPL*, *NIPBL1*, *NPM1*, *NRAS*, *PHF6*, *PTPN11*, *RAD21*, *RIT1*, *RUNX1*, *SETBP1*, *SF3B1*, *SMC1A*, *SMC3*, *SRSF2*, *STAG2*, *TET2*, *TP53*, *U2AF1*, *WT1*, and *ZRSR2*. Variant allele frequency (VAF) was significant over a threshold of 1%. Variants between 0% and 1% were confirmed with another NGS technology with a library preparation using the Haloplex Target Enrichment System (Agilent Technologies) and run on MiSeq (Illumina). The variant interpretation was performed according to their absence in public databases of polymorphisms (especially GnomAD) and their status in our “in-house” database of more than 8,000 samples validated (including AML samples from the Acute Leukemia French Association and MDS samples from the Groupe Français des Myélodysplasies)

Whole-blood samples were stained with dot plot CD45-KO (clone J33) to isolate the monocyte population. The three subsets of monocytes are gated with the combination CD16/CD14.

## Results

A 25-year-old woman was hospitalized for progressive abdominal pain. Regarding her medical history, Budd–Chiari syndrome was diagnosed at the age of 14 years, which led to long-term treatment with vitamin K antagonists. During this episode, the search for hereditary thrombophilia (factor V Leiden mutation, prothrombin gene mutation, protein C and S deficiency, antithrombin-III deficiency) or acquired thrombophilia (antiphospholipid syndrome, paroxysmal nocturnal hemoglobinuria) was negative. The search for *JAK2* mutation was negative. Bone marrow examination was not performed because the cells in the blood count were normal.

**Figure 1:**
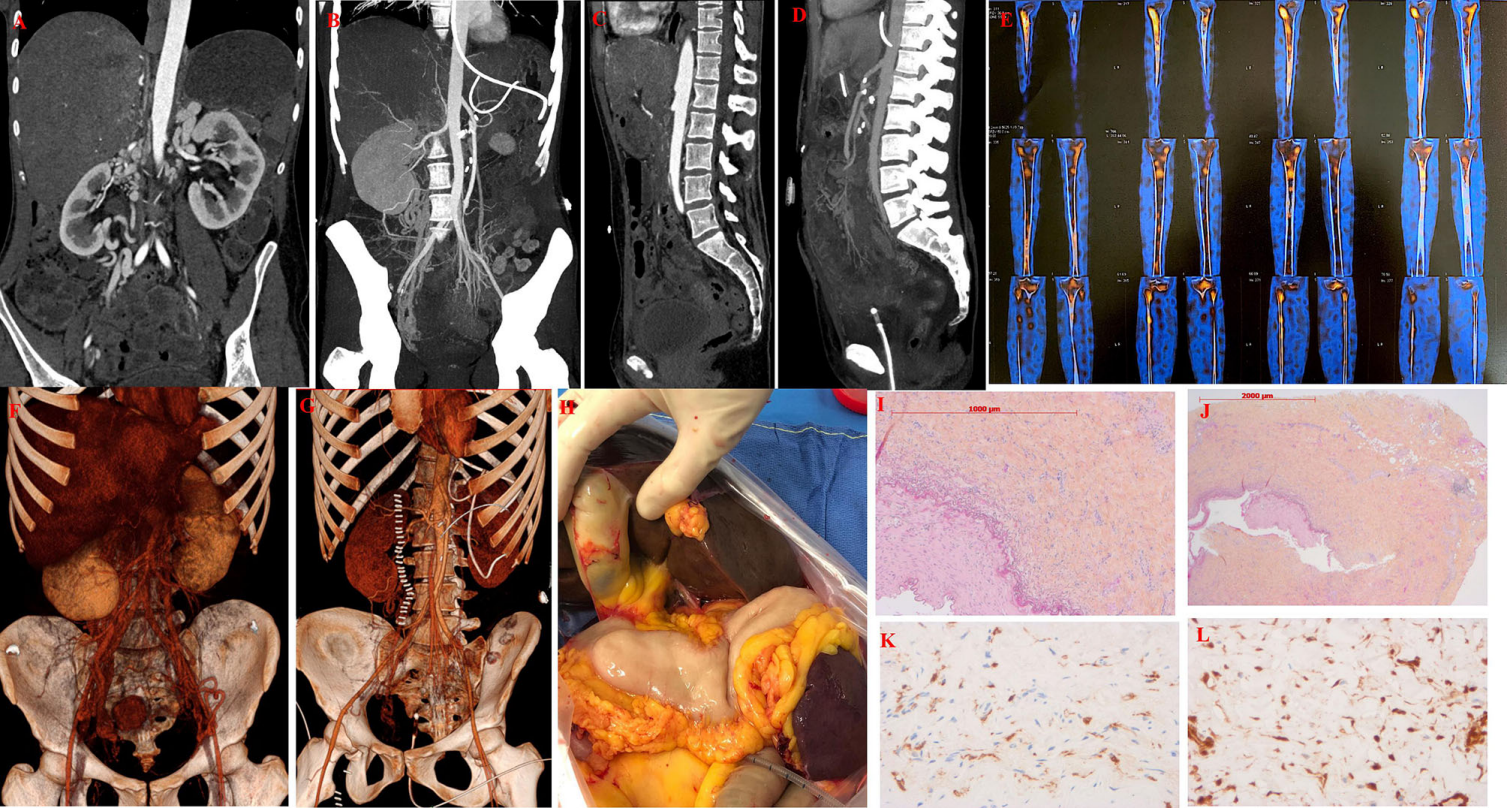
Clinical, radiological, and histological features of Erdheim–Chester Disease. **(A)** Sagittal computed tomography of the patient showing severe stenosis of superior mesenteric artery responsible for mesenteric ischemia in a patient with ECD. **(B)** Sagittal computed topography of the patient after thrombectomy and stenting of superior mesentery artery. **(C)** Coronal view of severe stenosis of superior mesenteric artery responsible for mesenteric ischemia in a patient with ECD. **(D)** Coronal view after thrombectomy and stenting of superior mesentery artery. **(E)** Bone scintigraphy showing radiotracer uptake on long bones characteristic of ECD. **(F)** Maximum intensity sagittal and angio3D projection showing vascular stenosis of coeliac trunk and superior mesentery artery. **(G)** Maximum intensity sagittal and angio3D projection demonstrates extensive digestive. **(H)** Maximum intensity sagittal and angio3D projection after digestive removal and vascular stenting. **(I)** Tissue biopsy of mesentery artery showing fibrosis of vessels adventitia without signs of vasculitis (×100). **(J)** Same sample showing adventitia fibrosis without signs of vasculitis (×200), **(K)** Same samples showing tissue infiltration with multinucleated histiocytes with CD163 (brown staining) expression on immunostaining.(HES; immunohistochemistry, ×200) consistent with ECD. **(L)** Same samples showing tissue infiltration with multinucleated histiocytes expressing phosphor-Erk (brown staining) HES; immunohistochemistry, (200×).

The current medical history started with recurrent abdominal pain responsible for weight loss (13 kg in 4 months). The physical examination was unremarkable. Biological tests showed an inflammatory syndrome (C-reactive protein, 300 mg/L). CT scan showed mesenteric ischemia secondary to the superior mesenteric artery sheath and celiac trunk (**Figure 1**) associated with bilateral nephromegaly. The patient underwent thrombectomy of the superior mesenteric artery associated with a secondary stent graft with successful reimplantation on the abdominal aorta associated with heparin-anticoagulation therapy. Despite the anticoagulation treatment, she presented with extensive digestive ischemia that required partial surgical removal of the jejunum. Investigations for hereditary or acquired thrombophilia were again negative. Mesentery artery biopsy showed normal intima and media, without atherosclerosis or vasculitis. The arterial lumen was normal without any sign of recent or ancient thrombosis. The adventitia contained abundant fibrosis with a mild leukocyte infiltration including lymphocyte and histiocyte but only rare plasma cells. The immunohistochemical analysis showed CD68^+^, CD1a^−^ histiocytes with strong phospho-ERK expression (**Figure 1**). NGS analysis on the biopsy tissue showed no mutation in the MAP-kinase pathway gene. Extensive analysis of the bone marrow showed no dysplasia and no reported mutations in clonal hematopoiesis. Thus, there was no evidence of hematological malignancy, solid tumor (on body CT scan), and infectious or rheumatological disease (connective tissue disease, vasculitis).

Technetium bone scan showed radiotracer uptake by long bones highly suggestive of ECD (**Figure 1**).

**Figure 2:**
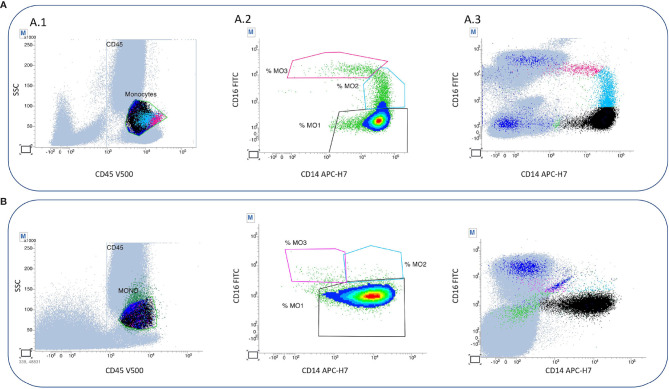
Flow cytometry analysis of monocyte subset on peripheral blood samples of a patient with ECD. **(A)** Flow cytometry analysis of monocyte subset in ECD remission. A.1 Monocyte population (green) is gated in dot plot SSC/CD45, and the zone is purified residual population (blue) by exclusion of T and NK lymphocytes with expression of CD7, granular cells with expression of CD16^+^ and CD14^+^) and B lymphocytes without CD16^-^ but with expression of CD24^+^. A.2 The three subtypes of monocytes are gated with the combination CD16/CD14: classical monocytes are MO1 (black) CD16^−^CD14^+^ representing 92.9% of total monocytes. Intermediate monocytes are MO2 (blue) CD16^+^ CD14^+low^ and represent 5.7% of total monocyte. Non-classical monocytes are MO3 (pink) expressing CD16^+^ without CD14 and representing 1.2% of total monocytes. A.3 In order to define the subtype, the gate is positioned with the help of all cells. **(B)** Flow cytometry analyses of monocyte subset in ECD flare The three monocytes subsets are gated with the combination CD16/CD14: classical monocytes are MO1 ((black) CD16^−^ CD14^+^ representing 97.5% of total monocytes. Intermediate monocytes are MO2 (blue) CD16^+^ CD14^+low^ representing 0.7% of total monocytes. Non classical monocytes are MO3 (pink) CD16^+^ CD14^-^ representing 1% of total monocytes. CD45^V500^, CD7^V450^, CD16^FITC^, CD24^PE^, CD14^APC-H7^, and CD56^PC7^. BC, Beckman Coulter Immunotech, Miami, FL, USA; BD, BD Biosciences, San Jose, CA, USA; V500, Horizon V500; APC-H7, allophycocyanin-H7; PC7, phycoerythrin-cyanin-7; PE, phycoerythrin; V450, Horizon V450; FITC, fluorescein isothiocyanate. Peripheral blood mononuclear cells (PBMC) were stained with CD15, CD 14, CD13 PE-CF594, CD33, CD34, and CD45 KO, and monocytes subsets were sorted. PBMC were centrifugated on a microscope slide, dried at room temperature for 30 min, and stained using May–Gruenwald–Giemsa stain. Monocyte population is gated in dot plot SSC/CD45, and the zone is purified of residual population by exclusion of T and NK lymphocytes with expression of CD7, granular cells with expression of CD16^+^ and CD14^+^ and B lymphocytes without CD16^−^ but with expression of CD24^+^.

**Figure 3:**
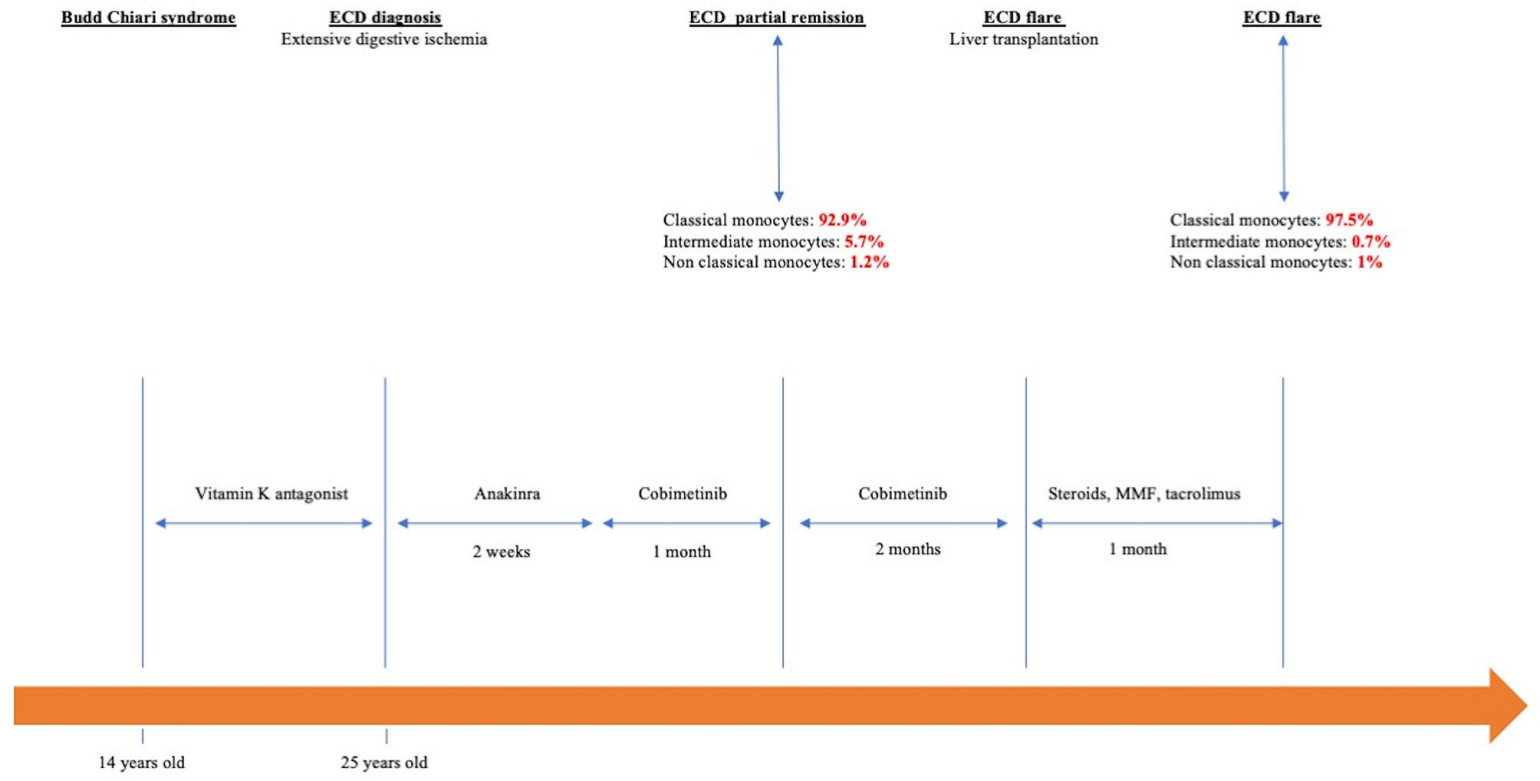
Chronological history of the patient from Budd–Chiari syndrome to ECD.

In the early postoperative period, the patient received an interleukin-1 receptor antagonist (Anakinra 100 mg/day) for 2 weeks, followed by an *MEK* inhibitor (Cobimetinib) 40 mg/day (20 mg twice daily, for 21 days of a 28-day cycle). Cobimetinib induced partial metabolic remission with decreased radiotracer uptake by long bones on ^18^fluorodeoxyglucose PET-CT. After Cobimetinib induced partial metabolic remission, monocyte subset analyses showed 92.9% “classical” MO1 monocytes (CD14^++^CD16^−^), 5.7% “intermediate” MO2 monocytes (CD14^+^CD16^+^), and 1.2% “non-classical” MO3 monocytes (CD14^low^CD16^+^). The total white blood cell count was 7.5 G/L. The monocyte count was 0.62 G/L or 7% of the total white blood cell count. Unfortunately, the patient developed a new celiac trunk thrombosis causing acute liver failure that required an emergency liver transplant. Prevention of graft rejection included high-dose steroids (500 mg/day in pulses for 3 days and then 1 mg/kg/day followed by a gradual decrease in steroids), a calcineurin inhibitor (tacrolimus, 0.2 mg/kg/day), mycophenolate mofetil (2 g/day), and basiliximab (20 mg/day for 4 days after transplantation). Cobimetinib was stopped after transplantation. One month later, despite immunosuppressive agents and curative anticoagulant therapy, the patient developed new thrombotic events and immunosuppressant-related infections and died. During this ECD flare, the blood cell count reached a total of 8.87 G/L, with a monocyte count of 0.33 G/L (3.7% of total white blood cells). Monocyte subset analyses showed an increase in “classical” monocytes (97.5%) and a decrease in “nonclassical” monocytes (1%) with 0.7% “intermediate” monocytes (**Figure 2**). The entire clinical course of the patent is reported in **Figure 3**.

## Discussion

We present a case report evaluating the correlation between monocyte subset and disease activity in the early follow-up of ECD. Papo et al. studied the distribution of monocyte subsets in patients with ECD without monocytosis (13). Patients with active ECD had an increase in “classical” monocytes and a decrease in “non-classical” monocytes compared with treated patients (considered to have controlled disease). This distribution resembles that of patients with CMML (16).

This distribution seems logical since most mutations in the MAP-kinase pathway genes occur in CD14^+^ monocytes (10, 11). Based on this consideration, we believe that uncontrolled expansion of CD14^+^ cells could be a cornerstone of the pathogenesis of histiocytosis. Even though mutated CD14^+^ monocytes represent a marginal part of blood cells, their expansion leads to classical monocytes and to the production of dendritic cells and homing tissue macrophages in histiocytic disorders. Our result regarding the level of classical monocytes in controlled ECD is comparable to the study of Papo et al. (13). Furthermore, our work reports the repeat analysis of monocyte subsets in a patient with ECD, and the expansion of classical monocytes appears to be associated with disease activity. CD14^++^CD16^low^ monocyte expansion was primarily assessed in CMML when the monocyte count was >1 G/L (16). CMML is a hybrid myelodysplastic/myeloproliferative disease that may overlap with histiocytosis (17, 18) and whom sometimes involve the MAP-kinase pathway (19, 20). By analogy with the CMML flare in proliferative patients, we believe that the expansion of CD14^+^ monocytes is associated with the flare in ECD, independent of the monocytes count in histiocytosis.

It should be noted that non-classical monocytes (CD14^low^, CD16^++^) are involved in the wound healing process (21) and promote endothelial adhesion of neutrophils *via* TNF-alpha secretion (22). In patients with atherosclerosis, their decrease correlates with coronary plaque progression (23). Our case calls into question the role of “non-classical” monocytes as potential markers of vascular disease because their decrease was associated with thrombosis.

This case also reports an aggressive vascular presentation of ECD that led to organ transplantation at an early age. ECD usually occurs in middle-aged patients (5, 6, 9), but pediatric patients have been reported previously (24). The current observation is that ECD is not a disease of the heart. In the current observation, Budd–Chiari syndrome at 14 years of age may be the first vascular event related to ECD. ECD should be sought in unusual site thrombotic events, including Budd–Chiari syndrome. On the other hand, transplantation has been reported only twice in patients with ECD-related organ failure (25, 26). It was associated with favorable outcomes in patients with ECD-related organ failure. It was associated with favorable outcomes in both cases, but the ECD was in remission at the time of transplantation, which was not the case in our patient. Neither immunosuppressive drugs (calcineurin inhibitors, mycophenolate mofetil) nor *MEK* inhibitor induced remission of ECD in this patient. The fact that the disease is not controlled by a *MEK* inhibitor in non-mutated ECD patients calls into question another dominant activation pathway rather than MAP-kinase activation (possibly PI3K/AKT/mTOR) (27). Other pathways have recently been described in patients with histiocytosis, (28, 29) suggesting new therapeutic targets in refractory patients. Inhibition of *RANKL* may be a promising therapeutic approach because *RANKL* is expressed in active ECD lesions along with p56 *NF-kB* activation (29, 30). *CSF1-R* inhibitor can also suppress dendritic cell differentiation and migration in histiocytosis (31). This approach may decrease the local tumor infiltrate and may reduce disease activity in refractory patients.

Treatments may interfere with monocyte assays; patient-administered immunosuppressive drugs (calcineurin inhibitors, mycophenolate mofetil) have no effect on monocyte subsets (32), but steroids in renal transplant patients may increase the ratio of classical monocytes and decrease that of non-classical monocytes (32). Indeed, the subset distribution results may have been biased by steroid use. However, in the cohort of transplanted patients, the change in monocyte distribution was not associated with clinical events. The change in the classical/non-classical monocyte ratio resembles what is observed in uncontrolled vascular disease. From our point of view, we cannot exclude a unfavorable role of steroid use in patients with ECD.

The main strength of our presentation is the use of monocyte subset analysis in two consecutive samples from an ECD patient, which shows an increase in classical monocytes correlated with the flare-up. This presentation may provide reassurance of the central role of monocytes in the pathophysiology of histiocytosis. The main limitation of the study is a case report, and the results need to be validated in a prospective cohort of patients. Thus, the functional role of monocyte subsets in histiocytosis is not fully elucidated, and basic science studies are mandatory to compare monocyte polarization to major pathologies described in the literature and broadly explore their pivotal role in histiocytosis.

Nevertheless, this bioassay may be useful in specific situations where ^18^FDG PET may be misleading (infection, postoperative period). Physicians may repeat it more frequently than ^18^FDG PET in patients with ECD.

In conclusion, this case supports the hypothesis that monocyte subset analysis can be a simple tool to assess ECD activity. However, further studies are needed to confirm this hypothesis.

## Data Availability Statement

The raw data supporting the conclusions of this article will be made available by the authors, without undue reservation.

## Ethics Statement

Written informed consent was obtained from the individual(s) for the publication of any potentially identifiable images or data included in this article.

## Author Contributions

JR collected the data and wrote the initial draft. J-FE confirmed histological features of ECD. AR and FG-O performed the cytometry analysis. All authors participated in the primary care of the patient and provided clinical, radiographic, and histological data of the patients. All authors contributed to the article and approved the submitted version.

## Conflict of Interest

The authors declare that the research was conducted in the absence of any commercial or financial relationships that could be construed as a potential conflict of interest.

## Publisher’s Note

All claims expressed in this article are solely those of the authors and do not necessarily represent those of their affiliated organizations, or those of the publisher, the editors and the reviewers. Any product that may be evaluated in this article, or claim that may be made by its manufacturer, is not guaranteed or endorsed by the publisher.
